# Overexpression of KMT9α Is Associated with Aggressive Basal-like Muscle-Invasive Bladder Cancer

**DOI:** 10.3390/cells12040589

**Published:** 2023-02-11

**Authors:** Florestan J. Koll, Eric Metzger, Jana Hamann, Anna Ramos-Triguero, Katrin Bankov, Jens Köllermann, Claudia Döring, Felix K. H. Chun, Roland Schüle, Peter J. Wild, Henning Reis

**Affiliations:** 1 Department of Urology, University Hospital Frankfurt, Goethe University, Theodor-Stern-Kai 7, 60590 Frankfurt am Main, Germany; 2 Frankfurt Cancer Institute (FCI), University Hospital, Goethe University, Theodor-Stern-Kai 7, 60590 Frankfurt am Main, Germany; 3 University Cancer Center (UCT) Frankfurt, University Hospital, Goethe University, Theodor-Stern-Kai 7, 60590 Frankfurt am Main, Germany; 4 Klinik für Urologie und Zentrale Klinische Forschung, Klinikum der Albert-Ludwigs-Universität Freiburg, 79106 Freiburg, Germany; 5 Deutsches Konsortium für Translationale Krebsforschung (DKTK), 79106 Freiburg, Germany; 6 Dr. Senckenberg Institute of Pathology, University Hospital Frankfurt, 60590 Frankfurt am Main, Germany; 7 Frankfurt Institute for Advanced Studies, 60438 Frankfurt am Main, Germany

**Keywords:** chemotherapy, histone methyltransferase, MIBC, molecular subtypes

## Abstract

Muscle-invasive bladder cancer (MIBC) is associated with limited response rates to systemic therapy leading to a significant risk of recurrence and death. A recently discovered histone methyltransferase KMT9, acts as an epigenetic regulator of carcinogenesis in different tumor entities. In this study, we investigated the presence and association of histological and molecular subtypes and their impact on the survival of KMT9α in MIBC. We performed an immunohistochemical (IHC) analysis of KMT9α in 135 MIBC patients undergoing radical cystectomy. KMT9α was significantly overexpressed in the nucleus in MIBC compared to normal urothelium and low-grade urothelial cancer. Using the HTG transcriptome panel, we assessed mRNA expression profiles to determine molecular subtypes and identify differentially expressed genes. Patients with higher nuclear and nucleolar KMT9α expression showed basal/squamous urothelial cancer characteristics confirmed by IHC and differentially upregulated KRT14 expression. We identified a subset of patients with nucleolar expression of KMT9α, which was associated with an increased risk of death in uni- and multivariate analyses (HR 2.28, 95%CI 1.28–4.03, *p* = 0.005). In conclusion, basal-like MIBC and the squamous histological subtype are associated with high nuclear KMT9α expression. The association with poor survival makes it a potential target for the treatment of bladder cancer.

## 1. Introduction

Bladder cancer (BCa) causes 213,000 deaths worldwide every year, and > 90% of cases are urothelial carcinoma [1]. Twenty-five percent of patients present with muscle-invasive bladder cancer (MIBC) at the time of diagnosis, which is associated with 5-year overall survival rates of approximately 50%. Despite radical surgical treatments and cisplatin-based chemotherapies, the recurrence rates are high [2]. The introduction of immune-checkpoint inhibitors in the treatment of MIBC and metastatic urothelial carcinoma has improved the survival and management of patients [3,4,5,6,7,8]. However, only a subset of patients responds to immunotherapies and/or are resistant to chemotherapies. This requires the development of novel biomarkers to stratify patients, the combination of therapies, and the exploration of further effective therapeutic approaches for advanced BCa.

Developments in genomic sequencing techniques have led to broad genomic analyses and advanced our knowledge regarding BCa genetics and biology. In addition to changes in the genome and transcriptome, epigenetic alterations, in explicit DNA methylation and histone modifications, are known to contribute to cancer development [9,10,11]. Epigenetic changes are early events in cancer genesis that modify gene expression without causing changes in the DNA sequences [11]. Mutations in genes remodeling histone methyltransferases and demethylases are very frequent in BCa and occur in 48% and 36% of MIBC patients, respectively [11,12]. Histone methyltransferases conduct the transfer of a methyl group to lysine or arginine residues of histones and can regulate cancer-related genes contributing to cell proliferation, invasion, and epithelial–mesenchymal transition [13,14]. Thus, histone methyltransferases, as well as demethylases, are increasingly explored as potential biomarkers and therapeutic targets in BCa and other entities [11,15].

A newly discovered histone lysine methyl transferase named lysine methyl transferase 9 (KMT9) has recently been described [16]. KMT9 is a heterodimeric enzyme assembled of KMT9α (also known as N6 adenine–specific DNA methyltransferase 1 (N6AMT1) or C21orf127) and KMT9β (also known as TRMT112). KMT9 monomethylates lysine 12 of histone H4 (H4K12me1). In prostate cancer cells, KMT9 is localized at promoters of genes involved in the cell cycle and controls their proliferation. Of note, the knockdown of KMT9 changes the proliferation of castration-resistant prostate cancer cells in vivo and in vitro [16]. Baumert et al. showed the expression of KMT9α and -β in lung cancer cells, which was associated with poor survival in lung cancer patients. Depletion of KMT9α affected genes involved in the cell cycle, apoptosis, and proliferation and inhibited the proliferation of lung cancer cells [17]. In colorectal cancer, KMT9α is involved in carcinogenesis by controlling cancer stem cells and has been proposed as a therapeutic target [18].

The function and presence of KMT9α in BCa are yet undefined. Here, we investigated the presence, relevance, and impact on the survival of KMT9α in urothelial MIBC.

## 2. Materials and Methods

### 2.1. Cohort

Tissue samples and patient data used in this study were provided by the University Cancer Center Frankfurt (UCT). Written informed consent was obtained from all patients, and the study was approved by the institutional review boards of the UCT and the ethical committee at the University Hospital Frankfurt (project number: SUG-6-2018 and UCT-53-2021), which was conducted according to local and national regulations and according to the Declaration of Helsinki.

A total of 145 formaldehyde-fixed paraffin-embedded (FFPE) tissue samples from patients with MIBC treated at the department of Urology, University Hospital Frankfurt from 2010–2020 were retrieved from the archive of the Dr. Senckenberg Biobank of the Dr. Senckenberg Institute of Pathology.

Clinicopathological and follow-up data were gathered from medical charts and records of the University Cancer Center.

Histopathology of all cases was rereviewed by two genitourinary pathologists according to current WHO criteria [19]. Histological subtypes were reported if at least 10% of the tumor showed subtype histology, including pure and mixed tumors.

### 2.2. Immunohistochemical (IHC) Analysis

The construction of the tissue microarray (TMA) has been described before [20]. Hematoxylin and eosin (H&E) staining was performed on a Tissue-Tek Prisma Plus staining device (Sakura Finetek, Torrance, CA, USA). IHC-analyses of p53 (Clone DO-7, Ref. GA616, Dako/Agilent, Santa Clara, CA, USA), KRT5/6 (Clone: D5/16 B4; Dako/Agilent, Santa Clara, CA, USA), and GATA3 (Clone: L50-823; ready-to-use kit; Cell Marque, Rocklin, CA, USA) were conducted using the DAKO Omnis staining system (Agilent, Santa Clara, CA, USA) with the DAKO FLEX-Envision Kit (Agilent) according to manufacturer’s instructions. Staining of KMT9α (#27630, lot 20062017, produced and provided by Schüle Lab) and KRT14 (Clone: Poly9060; 1:10000; BioLegend Inc., San Diego, CA, USA) were performed manually.

Quantitative and semiquantitative analysis of IHC was annotated by genitourinary pathologists. For KMT9α, we determined the percentage of nuclear-positive tumor cells. Nucleolar KMT9α positivity was observed when more than 10% of tumor cells showed clear nucleolar KMT9α expression. TMA cores with either an absence of representative tumor tissue or the presence of staining artifacts were excluded from the analysis.

Abnormal immunohistochemical p53 expression (null type or overexpression) was used as a surrogate marker for mutations in the *TP53* gene. p53 expression was scored according to the percentage of clear nuclear-positive tumor cells (0% = null type, 1–50% = wild type, and > 50% = overexpression) [21,22,23].

### 2.3. RNA-Isolation and Molecular Subtype Calling

We took a 1mm punch from FFPE blocks of a representative tumor area with ≥ 50% tumor content, isolated the RNA with the “truXTRAC FFPE total NA Kit-Column” (Covaris, Woburn, MA, USA), and measured the RNA concentration using the QuantiFluor RNA System (Promega, Madison, WI, USA) according to the manufacturer’s protocol. The mRNA expression of 19,398 mRNA targets was determined using the HTG Transcriptome Panel (HTG Molecular Diagnostics, Tuscon, AZ, USA) on an Illumina NextSeq 550 system (Illumina, San Diego, CA, USA), as described before [20]. Gene counts were normalized using median normalization and log2-transformed for further analysis. Molecular subtypes of MIBC were determined using the R-based consensus MIBC classification tool and the Bioconductor package for R [24]. Differentially expressed genes were identified using DESeq2 (version 1.30.1) [25]. *p* value  <  0.001 and expression fold-change  > 2 or  < (− 2) were considered statistically significant.

### 2.4. Statistical Analysis

For the survival analysis, we included patients with radical cystectomy. Patients with distant metastases or neoadjuvant chemotherapy were excluded from the analyses. We primarily assessed the overall survival (OS) as the endpoint, which was defined as the time interval between surgery and death.

The Kaplan–Meier method was used to estimate and illustrate survival probabilities. We used uni- and multivariable Cox’s proportional hazards models to estimate the hazard ratio (HR) and the corresponding 95% confidence interval for the survival. All tests were two-tailed; we used a significance level of α = 5%. Statistical analyses were performed using JMP (SAS Institute Inc., Cary, NC, USA) version 16.2.0 and R Studio (version 2022.02.3).

## 3. Results

### 3.1. KMT9α Expression in Normal Urothelium and Urothelial Cancer

**Figure 1:**
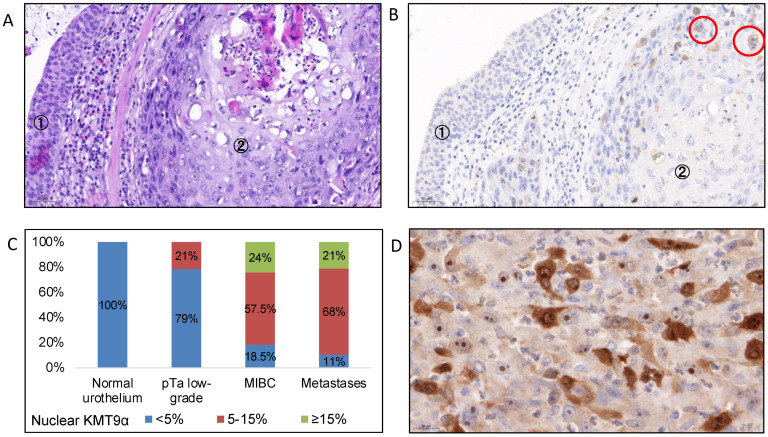
(**A**) HE-staining of normal urothelium (1) next to an invasive tumor with squamous histological subtype (2), magnification 200×. (**B**): IHC for KMT9α. Normal urothelium (1) with a slight cytoplasmatic background but without nuclear expression. The neighboring tumor (2) shows nuclear expression of KMT9α in 10% of tumor cells, magnification 200×. (**C**): Percentage of cases with low (<5%), intermediate (5–15%) and high (≥15%) nuclear KMT9α expression in “normal” urothelium (*n* = 8); urothelial pTa low-grade tumors (*n* = 14); MIBC (*n* = 135) and urothelial cancer metastases (*n* = 19). (**D**) Tumor with strong nuclear and nucleolar expression of KMT9α, magnification 630×.

Of the 145 tumor samples, 135 samples were evaluable for the immunohistochemical expression of KMT9α. In addition to the expression of KMT9α in 135 MIBC samples, we analyzed 8 normal urothelial samples without evidence of tumors, 14 pTa-low grade urothelial cancer samples, and 19 urothelial cancer metastases from lymph nodes (*n* = 16), lung (*n* = 1), and peritoneum (*n* = 2). The cytoplasmatic expression (% of positive tumor cells) of KMT9α was heterogenous between samples, but it was significantly lower in the metastases (mean 39% of cells, 95% CI 26–52%) compared to normal urothelium (mean 73% of cells, 95% CI 47–99) and the MIBC samples (mean 69%, 95% CI 64-74), (*p* = 0.01 and < 0.001, respectively). The nuclear expression of KMT9α was significantly higher in MIBC (mean 12.5%, 95% CI 11–14) and metastases (mean 12.5%, 10–16) compared to normal urothelium (mean 1%, 95% CI 0–2) and pTa-low grade tumors (mean 2%, 95% CI 1–4), *p* < 0.0001 (Figure 1). Overall, 101 (75%) patients showed nucleoli and 23 (17%) had KMT9α expression in the nucleolus (Figure 1D).

### 3.2. Patient Characteristics

Overall, we included specimens from 135 patients with MIBC [20]. The median age was 70 years (IQR 59–76). In total, 105 (78%) patients were male, and 30 (22%) were female. The clinicopathological details of the cohort are summarized in Table 1 and Table 2, stratified for patients with and without nucleolar expression of KMT9α and nuclear KMT9α < 5%, 5–15%, and ≥ 15%, respectively. The squamous histological subtype was significantly associated with nucleolar and high nuclear KMT9α expression, whereas the micropapillary histological subtype showed lower KMT9α expression values. Regarding the molecular subtypes, we noticed a significant association with the basal squamous subtype for nucleolar KMT9α expression. For higher nuclear KMT9α expression, such a trend towards the basal squamous subtype was also observed (Table 2).

### 3.3. Survival Analysis

We assessed the survival rates of 135 patients with adequate follow-up that received radical cystectomy in curative intent (cM0) without neoadjuvant chemotherapy and had available data for KMT9α expression. The median follow-up was 51 months (IQR 19–97 months). In this group, 40 patients received at least two cycles of adjuvant chemotherapy. The 12-month OS and disease-free survival (DFS) rates were 60% (95% CI 53–69) and 50% (95% CI 42–60), respectively.

**Figure 2:**
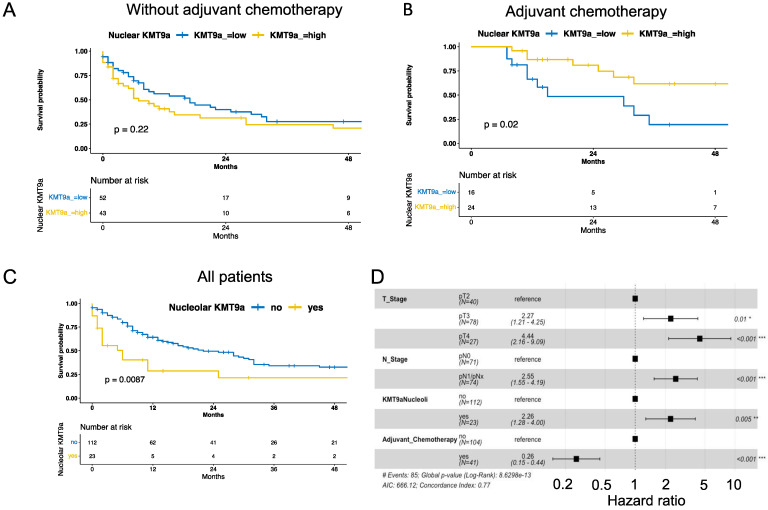
Kaplan–Meier curve for overall survival for patients with low and high (≥15% positive tumor cells) nuclear KMT9α expression stratified for patients with cystectomy only (**A**) and patients receiving adjuvant chemotherapy (**B**,**C**) Kaplan–Meier curve for all patients with and without nucleolar KMT9α expression. Nucleolar KMT9α positivity was called when more than 10% of tumor cells showed clear nucleolar KMT9α expression. *p*-values were calculated using log-rank test. (**D**) Forrest plot for the multivariate survival analysis adjusting for tumor and lymph node status, application of adjuvant chemotherapy, and nucleolar KMT9α expression. T_Stage = tumor stage; N_Stage = lymph node status; KMT9aNucleoli = Nucleoli positive for KMT9α expression. * *p* < 0.05; ** *p* < 0.01; *** *p* < 0.001.

Known predictive factors, such as tumor and lymph node stage as well as the application of adjuvant chemotherapy, were significantly associated with OS (Table 3 and Figure 2). In the total cohort, the nuclear expression of KMT9α was not associated with overall survival. After stratification for patients receiving only the cystectomy vs. patients receiving adjuvant chemotherapy, patients with a nuclear expression of KMT9α showed a significantly lower risk of death with adjuvant chemotherapy (HR 0.34, 95% CI 0.13–0.88, *p* = 0.03).

Patients showing the nucleolar presence of KMT9α (*n* = 23) had a significantly increased risk of death in the multivariate Cox regression model adjusting for tumor stage, lymph node status, and the application of adjuvant chemotherapy (Figure 2). Stratifying patients for the application of adjuvant chemotherapy, 17 patients with nucleolar KMT9α positive cells showed decreased survival without adjuvant chemotherapy (*p* < 0.001). For six patients receiving adjuvant chemotherapy and showing nucleolar KMT9α positive cells, no statistically significant difference in survival was observed (*p* = 0.85; Supplementary Figure S2).

### 3.4. KMT9α Expression Is Associated with Urothelial Basal Cell Characteristics

**Figure 3:**
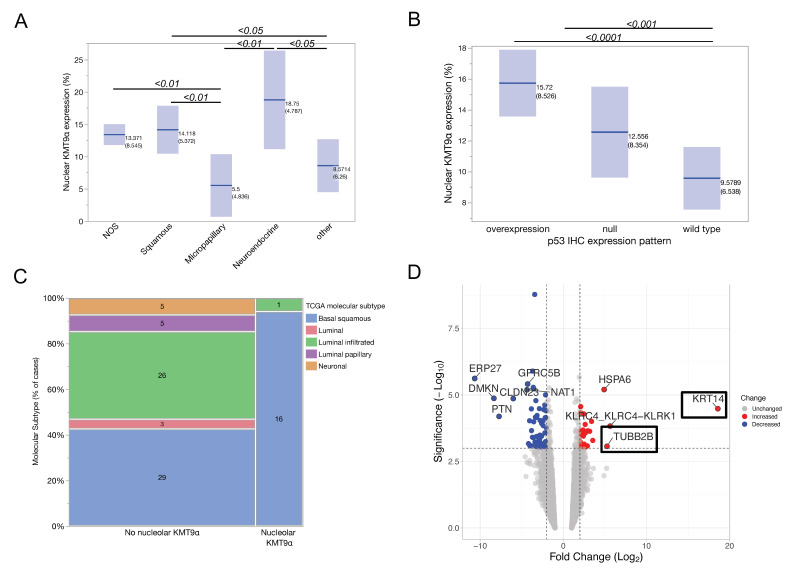
(**A**) Mean nuclear KMT9α expression was significantly associated with histological bladder cancer subtypes. Bars and numbers indicate the mean nuclear KMT9α expression and the standard deviations. Numbers above the bars indicate *p*-values (two-sided *t*-test) between groups. NOS (not otherwise specified), *n* = 91; squamous, *n* = 16; micropapillary, *n* = 10; neuroendocrine, *n* = 4; “Other” subtypes include glandular (*n* = 1), plasmacytoid (*n* = 4), lymphoepithelial (*n* = 3), nested (*n* = 1), sarcomatoid (*n* = 3), and giant cell (*n* = 1). (**B**) The nuclear expression of KMT9α (%) was significantly higher in patients with mutated *TP53* compared to wild type (*n* = 57). Mutation status was called when ≥50% of cells showed clear nuclear p53 overexpression (*n* = 50) or p53 null type (*n* = 27); *p* < 0.001. Values indicate mean nuclear KMT9α expression and standard deviations. IHC: immunohistochemistry. (**C**) Correlation between nucleolar KMT expressing tumors and molecular subtype according to the TCGA classification (*p* = 0.006), *n* = 85. (**D**) Volcano plot showing statistical significance (−log_10_ *p*-value) versus log_2_ expression fold change of genes comparing high (≥15%) vs. low (<5%) nuclear KMT9α expression. The 10 genes with highest fold change are annotated.

The expression of nuclear KMT9α showed an association with histological bladder cancer subtypes (*p* < 0.01, ANOVA). Tumors with squamous subtype (mean 14.1%, 95% CI 11– 17) and the “not otherwise specified” (NOS) subtype (mean 13.4%, 95% CI 11.6–15) had significantly higher nuclear KMT9α expression than tumors with micropapillary histology (mean 5.5 95% CI 2–9). Additionally, the neuroendocrine histological subtype showed significantly higher nuclear KMT9α expression (mean 18.75% 95% CI 11–26) than tumors with micropapillary (mean 5.5 95% CI 2–9), sarcomatoid (6.7%, 95% CI 0–13), and plasmacytoid (3.25%, 95% CI 0–11) subtypes (Figure 3A). IHC confirmed basal protein expression in tumors with high nuclear and nucleolar KMT9α expression: KRT5/6 was positive in 65% (15/23) of cases with nucleolar KMT9α and in 40% (44/110) of cases without nucleolar KMT9α (*p* = 0.03), and KRT14 was strongly positive in 48% (11/23) of cases with nucleolar KMT9α and 23% (25/109) of cases without nucleolar KMT9α (*p* = 0.01). The luminal marker GATA3 was associated with cases without nucleolar KMT9α expression: 56% (13/23) of cases with nucleolar KMT9α and 76% (83/109) of cases without nucleolar KMT9α (*p* = 0.05; Supplementary Figure S1).

We examined the immunohistochemical expression of p53, which is frequently mutated in bladder cancer leading to cell proliferation and genetic instability [12,26]. Fifty patients showed p53 overexpression, twenty-seven patients showed a complete absence of p53 expression (null type), and fifty-seven showed normal p53 expression (wild type). Patients with mutated *TP53* showed significantly higher nuclear KMTα expression (mean 14.4%; 95% CI 12.7–16.2 vs. 9.8 95% CI 7.7–11.8; two-sided *t*-test *p* < 0.001; Figure 3B). Sixty-five percent (15/23) of patients displaying nucleolar KMT9α had p53 overexpression, and thirteen percent (3/23) of patients had p53 null type, respectively (Chi^2^ *p* < 0.01; Supplementary Figure S3). An example of IHC-staining patterns for one case with nuclear and nucleolar KMT9α expression, p53, KRT5/6, and KRT14 overexpression is included in the Supplementary Material.

### 3.5. Molecular Subtyping and Differential Gene Expression

Eighty-five samples were available for molecular analysis to call molecular subtypes. Ninety-four percent (16/17) of patients with nucleolar KMT9α expression had the basal–squamous molecular subtype, and one nucleolar-positive case was classified as luminal infiltrated (Pearson Chi^2^ *p* = 0.006, Figure 3C). Molecular subtypes showed a significant association with IHC markers (Supplementary Figure S5)

Analyzing the differential gene expression of 11,572 genes between high (≥15%) and low (<5%) nuclear KMT9α expression, we identified *KRT14* as highly upregulated (fold change 18.59; raw P = 3.28 × 10^−5^; adj P = 1.39 × 10^−2^; Figure 3D). *KRT14* has been described before as a marker of basal cells with stem cell characteristics that represents the most primitive differentiation state of urothelial carcinoma [27,28,29]. Additionally, *TUBB2B* was upregulated in tumors with high KMT9α expression (fold change 5.25; raw P = 8.54 × 10^−4^; adj P = 6.66 × 10^−2^), which is considered a marker of small-cell/neuroendocrine-like urothelial carcinoma [30,31,32].

## 4. Discussion

In this study, we investigated the expression and significance of KMT9, a novel H4K12me1 histone methyltransferase, in a cohort of patients with muscle-invasive bladder cancer. High nuclear expression of KMT9α was found in a significant number of cases and was associated with basal squamous and neuroendocrine histological and molecular subtypes. Nucleolar KMT9α expression was an independent predictor of poor prognosis.

These findings are in line with previously published data regarding KMT9 in other malignancies. For lung cancer, the histone methyltransferase KMT9 was shown to regulate the proliferation and survival of small-cell lung cancer and NSCLC cells [17]. Similarly, Metzger et al. described KMT9α to regulate prostate tumor proliferation [16]. Furthermore, Berlin et al. proposed KMT9 as an important regulator of colorectal carcinogenesis using mouse models and human tumor organoids [18].

Nucleolar KMT9α expression was associated with decreased survival, especially in patients not receiving adjuvant chemotherapy (Figure S2). The nucleolus is the location of ribosomal biogenesis, which is often increased in cancer cells [33,34]. However, our data did not allow conclusions regarding the functions of KMT9 in the nucleolus.

Higher nuclear KMT9α expression and more KMT9α nucleolar positive cases were observed in patients with abnormal p53 expression, which is a surrogate marker for mutations in the *TP53* gene [23]. *TP53* mutations are considered a hallmark of high-grade urothelial cancer and have been described to occur in basal squamous and neuronal molecular subtypes in 61% and 94%, respectively, which is in line with our data (Figure S6) [12,24,35,36]. P53 is a tumor-suppressor and key mediator of stress signaling; thus, it is closely related to ribosomal biogenesis and nucleolar functions [33,34]. So far, the correlation between mutated *TP53* and nucleolar KMT9α expression was observed on a case level, but higher resolutions and functional analyses are required to uncover interactions between KMT9 and p53 on a single cell level.

Our results show that the expression of KMT9α is associated with basal gene and protein expression, leading to a squamous phenotype in many cases. This also translates into a survival detriment in patients with nucleolar KMT9α expression (Figure 2C). Interestingly, the neuroendocrine histological and molecular subtype also shows higher nuclear KMT9α expression but no nucleolar KMT9α. Patients with molecular basal and neuronal MIBC have also been shown to have the worst prognosis in other studies [12,24,37].

On the other hand, we found that patients with high nuclear KMT9α expression had a survival benefit when treated with adjuvant chemotherapy. The data to predict response to (mainly neoadjuvant) chemotherapy based on histological or molecular bladder cancer subtypes is controversial, and so it cannot be concluded whether a basal–molecular subtype of MIBC responds well to chemotherapy [37,38,39,40]. Biomarkers to stratify MIBC patients are required to select patients for the application of perioperative chemotherapy. However, KMT9α will need prospective validation as a predictive marker in a larger cohort.

In addition to their importance as predictive and prognostic markers, novel therapeutic strategies are required as sequenced or combined approaches to improve the survival rates of BCa patients. The high presence of alterations in genes involved in histone modifications and preliminary data make histone methyltransferases a promising target [11,15,41]. As an example, a phase 1/2 trial is testing Tazemetostat, an EZH2 inhibitor, in combination with the immune-checkpoint inhibitor pembrolizumab in patients with advanced urothelial carcinoma (https://clinicaltrials.gov: NCT03854474, accessed on 8 January 2023). Results regarding KMT9α generated in vitro, in xenograft tumors, and in organoids [16,18], together with the overexpression in aggressive MIBC, must be translated into an applicable drug to inhibit KMT9α that can be explored as a potential therapeutic approach.

The limitation of our study is the retrospective design, leading to a selection bias in patients receiving chemotherapy. Furthermore, the number of samples included, especially in the molecular analysis, is rather low. Thus, findings need to be reproduced in larger datasets. Our analysis relies on histology, IHC, gene expression, and clinical data and delivers important insights into the expression of KMT9α in a well-characterized cohort of MIBC patients. However, functional analyses explaining the observed effects in bladder cancer are pending and are important to further explore KMT9 as a therapeutic target for BCa.

## 5. Conclusions

In conclusion, KMT9α is highly expressed in bladder cancer cells with aggressive basal and neuroendocrine phenotypes. This needs to be translated into further exploration of KMT9α expression as a predictive marker for chemotherapy response in MIBC. Furthermore, as a histone, methyltransferase KMT9α is a promising target, and the effect of its inhibition should be explored in vitro and in vivo to treat bladder cancer.

## Figures and Tables

**Table 1:** Clinicopathological details of 135 patients evaluable for nucleolar KMT9α IHC. mRNA expression profiles to determine molecular subtypes were available for 85 patients. IQR = interquartile range; NOS = not otherwise specified.

		No KMT9α in Nucleolus (*n* = 112)	KMT9α in Nucleolus (*n* = 23)	*p*
Nuclear KMT9α expression	0–5%	22 (19%)	2 (9%)	0.02
	5–15%	68 (61%)	10 (44%)	
	≥15%	22 (20%)	11 (48%)	
Median age (IQR)		69 (59–77)	71 (57–76)	n.s.
Gender	Male	87 (78%)	18 (78%)	n.s.
	Female	25 (22%)	5 (22%)	
Max. tumor stage	pT2	32 (29%)	3 (12.5%)	n.s.
	pT3	56 (50%)	18 (75%)	
	pT4	24 (22%)	3 (12.5%)	
pN stage	pN0	55 49%)	9 (39%)	n.s.
	pN+ (*n* = 59)/pNx (*n* = 12)	57 (51%)	14 (61%)	
Histological subtype	NOS	77 (69%)	14 (61%)	0.05
	Squamous	9 (8%)	7 (30%)	
	Micropapillary	9 (8%)	1 (4%)	
	Neuroendocine	4 (4%)	0	
	Sarcomatoid	3 (3%)	0	
	Plasmacytoid	4 (4%)	0	
	Other (3 Lymphoepithelial, 1 Nested, 1 Glandular, 1 Giant cell)	5 (4%)	1 (4%)	
TCGA molecular subtype	Basal squamous	29 (43%)	16 (94%)	<0.01
	Luminal	3 (4%)	0	
	Luminal infiltrated	26 (38%)	1 (6%)	
	Luminal papillary	5 (7%)	0	
	Neuronal	5 (7%)	0	

**Table 2:** Clinicopathological details of 135 patients evaluable for nuclear KMT9α IHC. mRNA expression profiles to determine molecular subtypes were available for 85 patients. IQR = interquartile range; NOS = not otherwise specified.

		Nuclear KMT9α < 5% (*n* = 24)	Nuclear KMT9α 5–15% (*n* = 78)	Nuclear KMT9α ≥ 15% (*n* = 33)	*p*
Nucleolar KMT9α	no	22 (92%)	68 (87%)	22 (67%)	0.02
	yes	2 (8%)	10 (13%)	11 (33%)	
Median age (IQR)		67 (56–73)	70 (59–78)	71 (62–75)	n.s.
Gender	Male	18 (75%)	61 (78%)	26 (79%)	n.s.
	Female	6 (25%)	17 (22%)	7 (21%)	
Max. tumor stage	pT2	7 (29%)	21 (27%)	7 (21%)	n.s.
	pT3	11 (46%)	40 (51%)	22 (67%)	
	pT4	6 (25%)	17 (22%)	4 (12%)	
pN stage	pN0	14 (58%)	33 (42%)	17 (52%)	n.s.
	pN+ (*n* = 59)/pNx (*n* = 12)	10 (42%)	45 (58%)	16 (48%)	
Histological subtype	NOS	15 (65%)	49 (63%)	27 (82%)	<0.01
	Squamous	0	13 (17%)	3 (9%)	
	Micropapillary	4 (17%)	6 (8%)	0	
	Neuroendocine	0	2 (3%)	2 (6%)	
	Sarcomatoid	0	3 (4%)	0	
	Plasmacytoid	3 (13%)	1 (1%)	0	
	Other (3 Lymphoepithelial, 1 Nested, 1 Glandular, 1 Giant cell)	1 (4%)	4 (4%)	1 (3%)	
TCGA molecular subtype	Basal squamous	4 (29%)	24 (52%)	17 (68%)	n.s.
	Luminal	0	2 (4%)	1 (4%)	
	Luminal infiltrated	8 (57%)	15 (33%)	4 (16%)	
	Luminal papillary	1 (7%)	4 (9%)	0	
	Neuronal	1 (7%)	1 (2%)	3 (12%)	

**Table 3:** Univariate Cox regression model for overall survival. High nuclear KMT9α expression was defined as ≥ 15% positive tumor cells. Nucleolar KMT9α positivity was called when more than 10% of tumor cells showed clear nucleolar KMT9α expression.

Variable		Hazard Ratio	*p*
Gender	Female vs. male	1.36 (0.82–2.28)	0.2
Tumor stage	pT3 vs. pT2	2.32 (1.31–4.13)	0.004
	pT4 vs. pT2	4.78 (2.49–9.15)	<0.0001
Lymphnode stage	pN+/pNx vs. pN0	2.35 (1.51–3.65)	0.0001
Adjuvant chemotherapy	Yes vs. no	0.43 (0.26–0.73)	0.002
Nuclear KMT9α	High vs. low	0.88 (0.57–1.35)	0.6
Nucleolar KMT9α	Yes vs. no	2.09 (1.21–3.61)	0.009
Nucleoli present	Yes vs. no	1.16 (0.71–1.91)	0.5

## Data Availability

The data that support the findings of this study are available from the corresponding author upon reasonable request.

## Supplementary Materials

The following supporting information can be downloaded at: https://www.mdpi.com/article/10.3390/cells12040589/s1, Figure S1: Correlation between nuclear and nucleolar KMTα expressing tumors and IHC of KRT5/6, GATA3 and KRT14. Figure S2: Kaplan-Meier curve for overall survival for patients with cystectomy only and patients receiving adjuvant chemotherapy stratified for nucleolar KMT9α expression. Figure S3: IHC for KMT9α, p53, GATA3, KRT5/6 and KRT14 of one representative case. Figure S4: Association of nucleolar KMT9α expression and p53 expression pattern. Figure S5: Association of molecular subtypes with IHC-expression of KRT5/6, GATA3 and KRT14. Figure S6: Association of molecular subtypes with p53 IHC expression patterns.
