# Supplementary material for: Overexpression of KMT9α Is Associated with Aggressive Basal-like Muscle-Invasive Bladder Cancer
Source: Cells. 2023 Feb 11;12(4):589. doi: 10.3390/cells12040589 (PMC9954512; doi:10.3390/cells12040589)
Supplement: Supplementary file 1 [file cells-12-00589-s001.zip › cells-2181153-supplementary.pdf]

Overexpression of KMT9α is associated with aggressive basal-like muscle-invasive bladder cancer

Figure S1

A

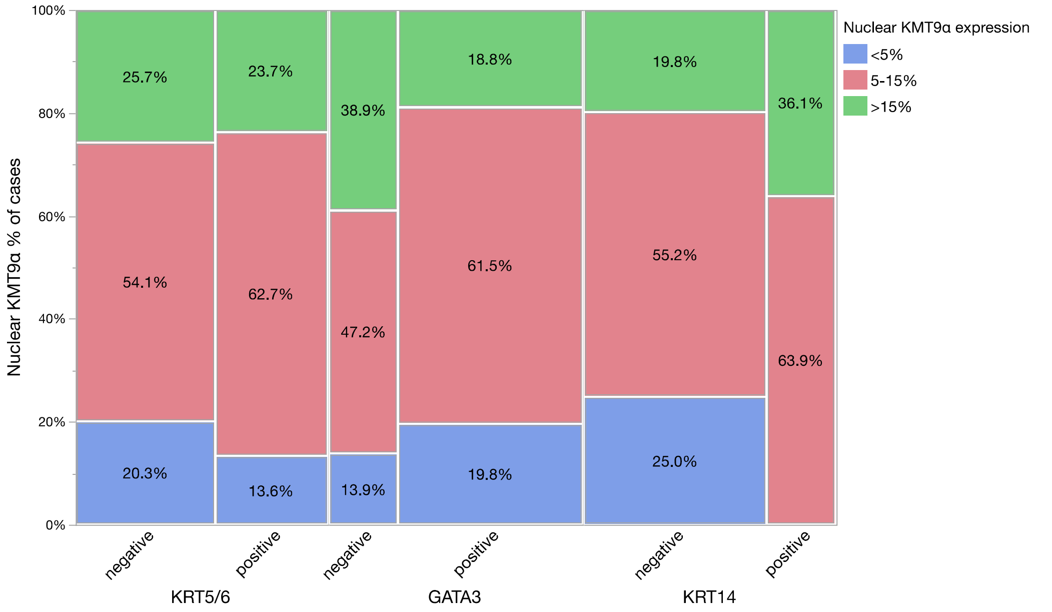

B

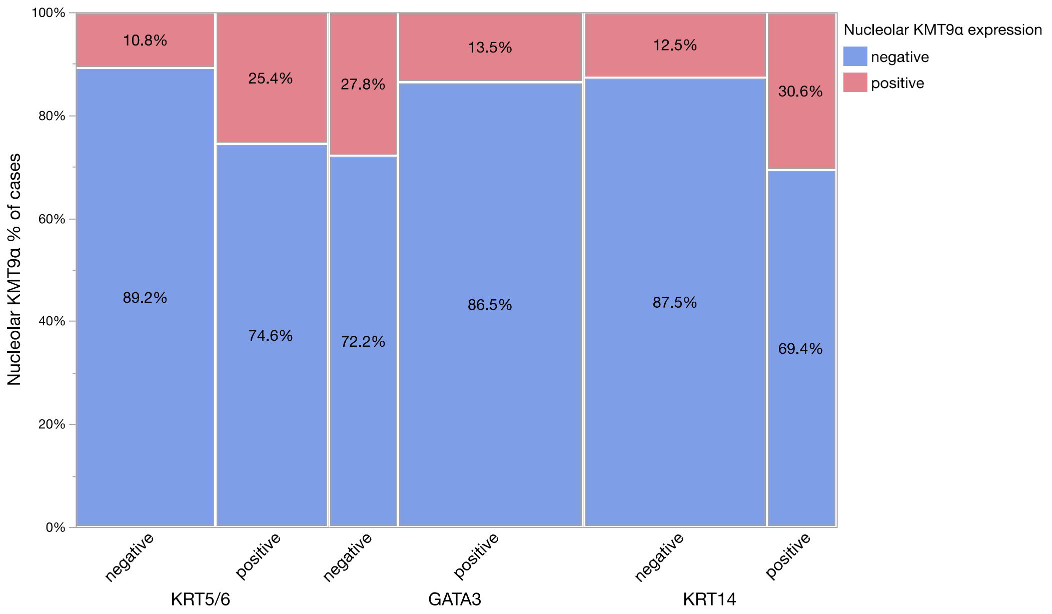

Figure S1: Correlation between nuclear (A) and nucleolar (B) KMTα expressing tumors and IHC of KRT5/6 (n=133), GATA3 (n=132) and KRT14 (n=132).

Figure S2

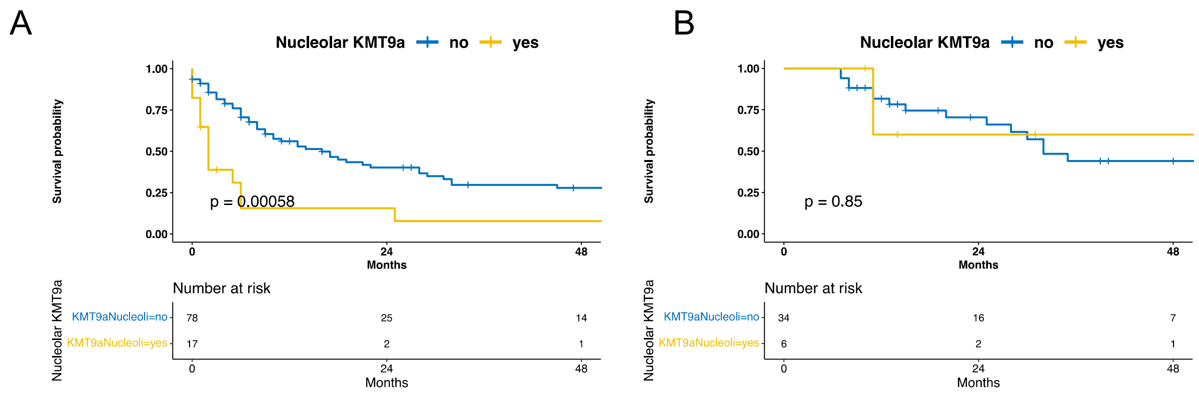

Figure S2: Kaplan-Meier curve for overall survival for patients with cystectomy only (A) and patients receiving adjuvant chemotherapy (B) stratified for nucleolar KMT9α expression.

Figure S3

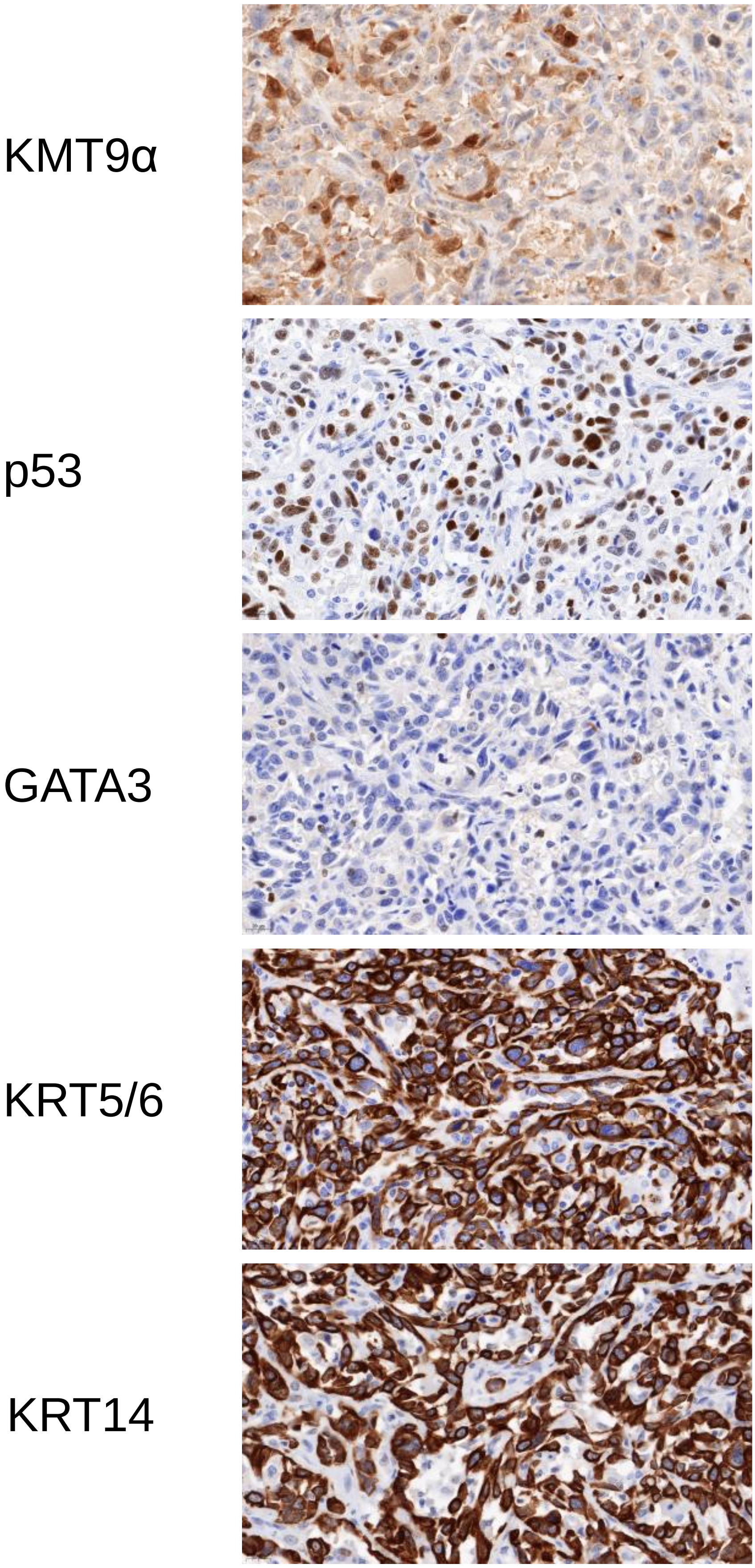

Figure S3: IHC for KMT9*α* (nuclear and nucleolar positive), p53 (overexpression), GATA3 (negative), KRT5/6 (positive) and KRT14 (positive) of one representative case; magnification 400x.

Figure S4

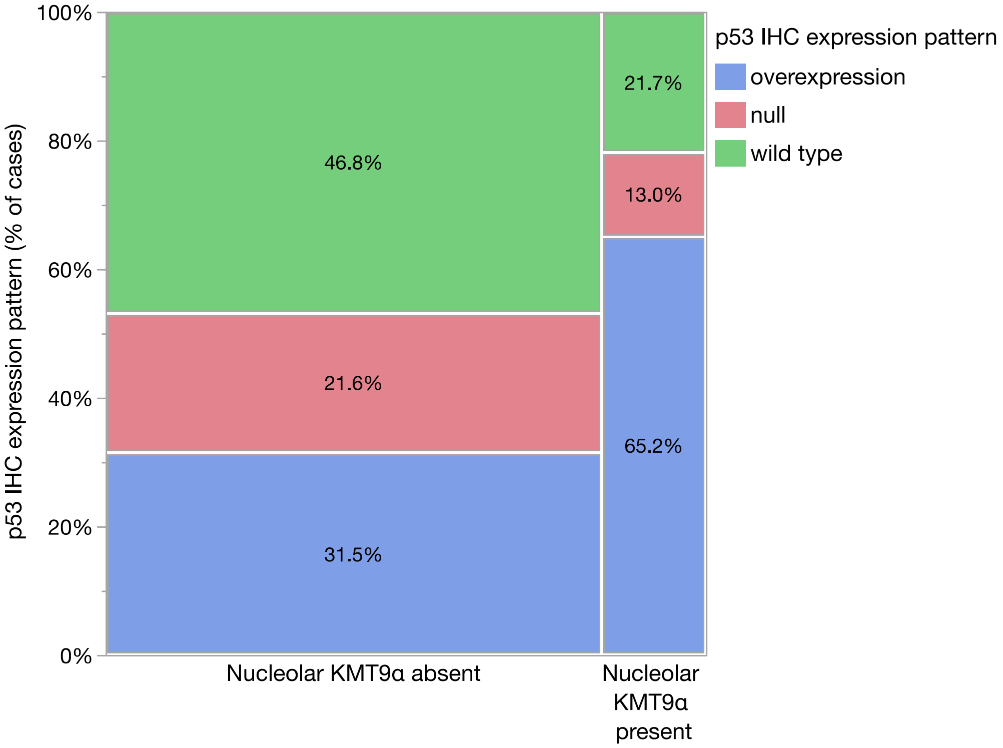

Figure S4: Association of nucleolar KMT9*α* expression and p53 expression pattern. Cases with nucleolar KMT9*α* expression showed p53 overexpression in 65% (15/23), null type in 13% (3/23) and wild type in 22% (5/23) of cases, respectively. This was significantly more, than in cases without nucleolar KMT9*α* expression (n=111), $p<0.01$.

Figure S5

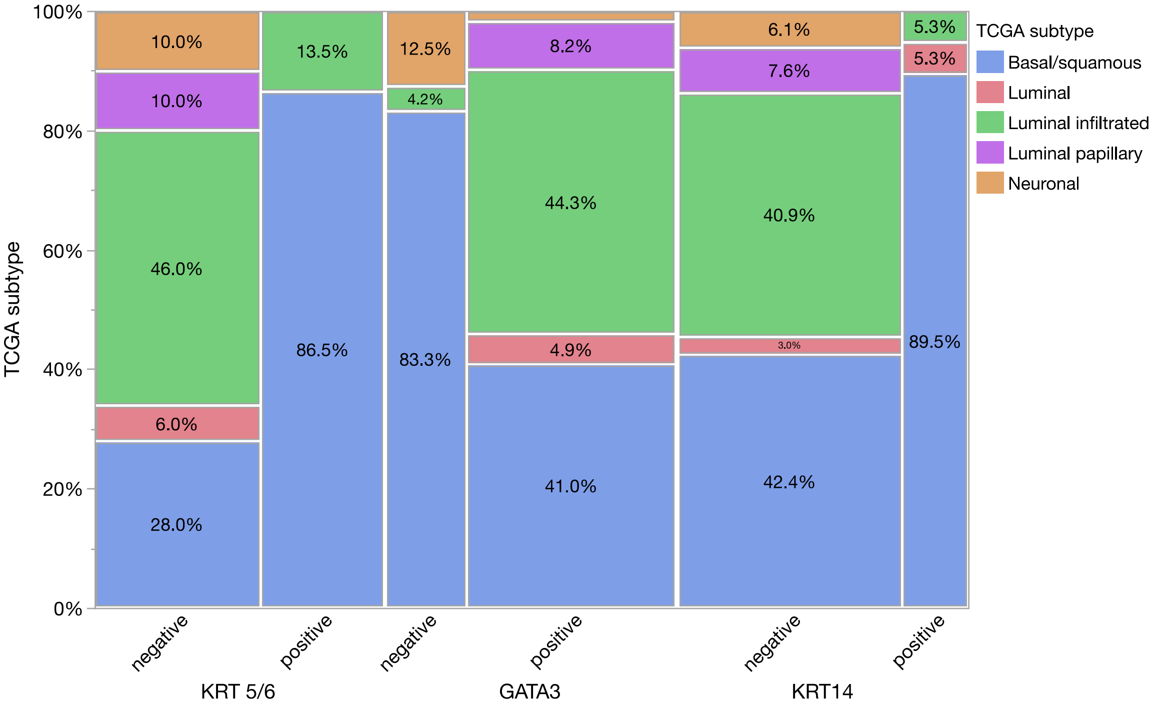

Figure S5: Association of molecular subtypes with IHC-expression of KRT5/6 ($p<0.0001$), GATA3 ($p=0.0002$) and KRT14 ($p=0.006$).

Figure S6

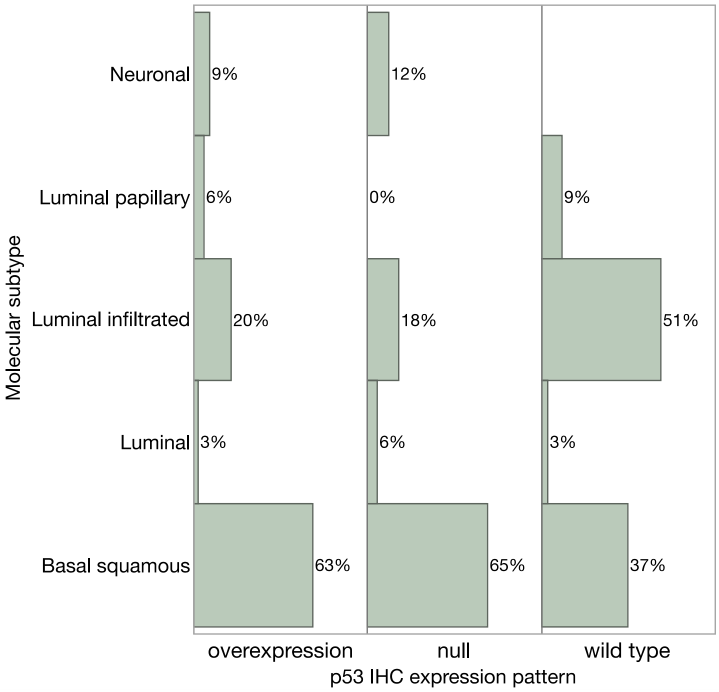

Figure S6: Association of molecular subtypes with p53 IHC expression patterns (Chi² p=0.06). The p53 expression was scored according to the percentage of clear nuclear positive tumor cells (0% = null type, 1–50% = wild type, and >50% = overexpression)
